# Flowers of Sulphur and Cream of Tartar and Magnesia in Dysentery

**Published:** 1842-05

**Authors:** 


### FLOWERS OF SULPHUR AND CREAM OF TARTAR AND MAGNESIA IN DYSENTERY.

Dr. Wm. Sherer, of Meade County, Kentucky, during the past summer and autumn, was induced to try the effect of this compound in a dysentery which prevailed in his neighborhood. He used equal parts of those medicines, mixed with cream, to the consistence of thin custard, of which he gave a desert spoonful every hour. After narrating the history of the first case, in which he he employed this mixture, he goes on to say:—

"I subsequently treated, with complete success, all the cases of dysentery to which I was called, (and I had some 15 or 20) in a similar manner, without giving any mercurial purges, except in one or two cases, where the tongue, by its smooth, dry, and glossy appearance, indicated a suspension of the secretions of the mucous membrane. In these cases, a few pills of blue mass restored the secretions to a healthy condition. I am inclined therefore to the belief that mercurial cathartics are not only unnecessary, in the treatment of dysentery, but perhaps injurious, except probably in those cases where the biliary organs are considerably deranged, which, by the bye, so far as my observations extend, is not very common. The localities in which the dysentery prevailed, were identical with those in which the bilious and congestive fevers also prevailed. It not unfrequently happened that different individuals of the same family would be attacked at the same time, some with dysentery, and others with bilious fever."

